# Supplementary material for: Excitation of coupled spin–orbit dynamics in cobalt oxide by femtosecond laser pulses
Source: Nat Commun. 2017 Sep 21;8:638. doi: 10.1038/s41467-017-00616-2 (PMC5608704; doi:10.1038/s41467-017-00616-2)
Supplement: Supplementary file 1 — Supplementary Information [file 41467_2017_616_MOESM1_ESM.pdf]

SI GUIDE

File Name: Supplementary Information
Description: Supplementary Figures, Supplementary Notes and Supplementary References.

File Name: Peer Review File
Description:

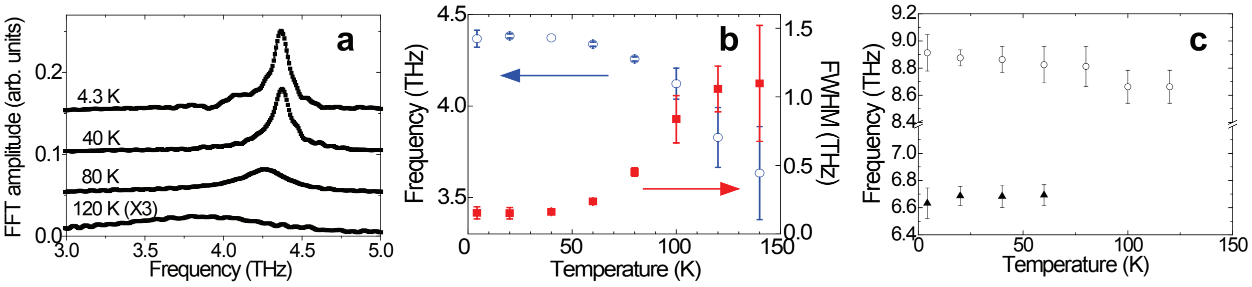

Supplementary Figure 1: **Temperature dependence of the magnon frequencies.** **a**, Fourier-transformed amplitude spectra of 4.4-THz magnon modes for various temperatures, when the azimuth of the pump light in LG is $\theta = 0°$. The measurement resolution was 66 GHz. **b**, Temperature dependence of the centre frequency and the full-width at half-maximum (FWHM) of the 4.4-THz magnon modes in Supplementary Figure 1a. The centre frequency of the 4.4-THz mode decreases with increasing temperature, indicating magnetic modes. **c**, Temperature dependence of the centre frequency of the 6.6-THz and 8.9-THz magnon modes. The frequencies of the 6.6-THz and 8.9-THz modes do not seem to drop to zero at the Néel temperature, but these modes have been assigned to magnon modes [1].

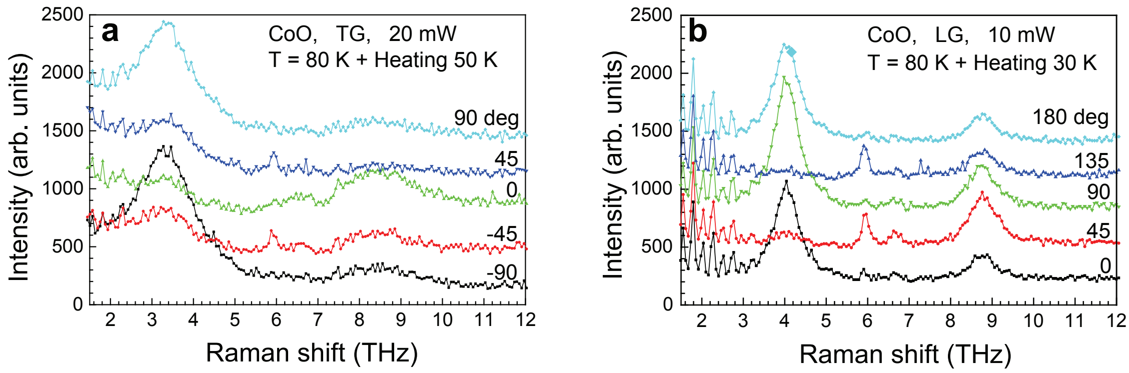

Supplementary Figure 2: **Spontaneous Raman scattering.** Confocal micro-Raman spectra in parallel Nicol geometry [2]. In each case, a single magnetic domain was chosen in which the magnetization was aligned **a**, in the sample plane (as in the TG) and **b**, out of the sample plane (as in the LG). The excitation wavelength was 532 nm. The spot diameter was 4 μm. The excitation powers were **a**, 20 mW and **b**, 10 mW, leading to laser heating of 50 and 30 K, respectively, in an ambient temperature of 80 K. The angle between the magnetization and incident polarization azimuth was varied **a**, from $-90°$ to $90°$ and **b**, from $0°$ to $180°$. In panel **a**, peaks were observable at 3.3 and 8.4 THz, which were assigned to the 4.4- and 8.9-THz magnon modes, respectively. The peak at 3.3 THz displayed maximum intensity at $-90°$ and $90°$, and its intensity was negligible at $0°$, suggesting it originated from the Γ_2 magnon mode with a G_4 term (Supplementary Equation (31)). The peak at 8.4 THz displays its maximum intensity at $0°$. This result indicates that the mode at 8.4 THz has different symmetry from that of the mode at 3.3 THz, in good agreement with our conclusion obtained in the pump-probe experiment in the TG. A peak corresponding to the 6.6 THz-mode was not visible, probably because the laser heating of up to 50 K diminished the intensity of this peak, as it is seen in Supplementary Figure 1. Another peak at 6.0 THz was also observed, but it was not found in any literature reports. Threfore, it may originate from impurities. In panel **b**, peaks are observable at 4.0, 6.6 and 8.8 THz, and are assigned to the 4.4-, 6.6- and 8.9-THz magnon modes, respectively. The peak at 4.0 THz shows maximum intensity at $0°$, $90°$ and $180°$, and is negligible at $45°$ and $135°$, suggesting it originated from the Γ_2 magnon mode with a G_4 term (Supplementary Equation (35)). The peak at 6.6 THz displays maximum intensity at $45°$ and $135°$, and is negligible at $0°$, $90°$ and $180°$, suggesting it arises from the Γ_1 magnon mode with the $(G_2 - G_3)$ term (Supplementary Equation (33)). The peak at 8.8 THz has different symmetry from those of the modes at 4.0 and 6.6 THz, in good agreement with our conclusion obtained from the pump-probe experiment in the LG. The peak widths are broader than the corresponding ones in the main text because of the temperature difference. Spike-like peaks below 3 THz are attributed to rotation modes in the ambient nitrogen gas atmosphere.

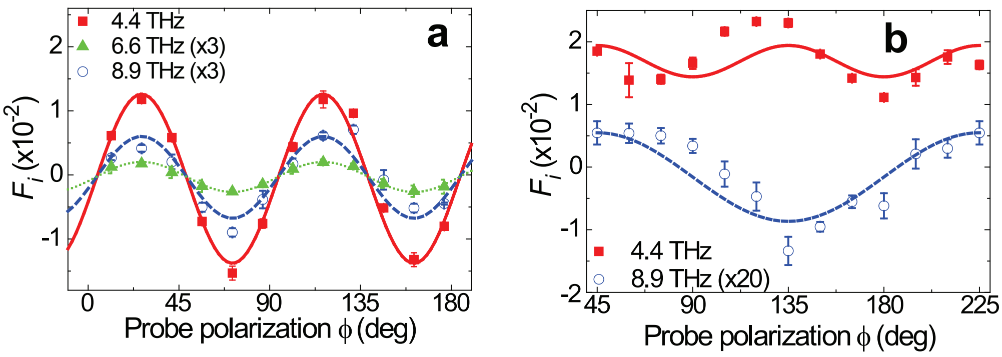

Supplementary Figure 3: **Modulation of the probe polarization by magnons. a**, Probe polarization dependence of F in TG for the 4.4-THz (solid line), 6.6-THz (dotted line) and 8.9-THz (dashed line) modes. The azimuthal angle of the linearly polarized pump light is fixed at $\theta = 94^\circ$ and that of the linearly polarized probe light ϕ is varied. The Faraday effect, the CME, magnetic circular dichroism and magnetic linear dichroism are all possible causes of the modulation of the probe polarization by magnon excitations. For the Faraday effect, F does not rely on the azimuth ϕ of the probe light. With the CME, F is proportional to $\sin(4\phi + \phi_1)$, where ϕ_1 is determined by the optic axis of magnetic birefringence. With magnetic circular dichroism, oscillations are not observed in F, regardless of ϕ. In magnetic linear dichroism, F is proportional to $\sin(2\phi + \phi_2)$, where ϕ_2 is determined by the optic axis of dichroism. The observed dependence was fitted by function $-\sin(4\phi + 4^\circ)$ for all oscillation modes of 4.4 THz, 6.6 THz and 8.9 THz, indicating that the magnons are observed by the CME of the probe light in the TG. **b**, Probe polarization dependence of F in the LG for the 4.4-THz (solid line) and 8.9-THz (dashed line) modes. The pump azimuth in the linear polarization is fixed at $\theta = 0^\circ$ and ϕ of the probe azimuth in the linear polarization is varied. F for the 4.4-THz mode is proportional to $7 + \cos 4\phi$. This rather weak dependence on ϕ means that the magnons are mainly observed with the Faraday effect of the probe light, and that in addition, the CME contributes to the signal to some extent. In contrast, F for oscillations of 8.9 THz is proportional to $-0.2 + \sin 2\phi$. This shows that the modulation of the probe polarization is caused by linear dichroism and the Faraday effect in the probe light.

Supplementary Note 1: Model Hamiltonian

Cobalt oxide CoO has two cobalt ions in each magnetic unit cell. The free Co^{2+} ion has the electronic structure $3d^7$ and its ground state corresponds to a ^4F term with orbital angular momentum $L_{\text{free}} = 3$ and spin $S = 3/2$. In the main (cubic) approximation, the crystal field splits this term into a few multiplets with the orbital triplet as the lowest state. Thus, unlike many transition-metal ions in compounds with cubic symmetry, which have a singlet ground state, the orbital angular momentum of the magnetic ion in CoO is only partially quenched. For the theoretical description of the magnetic ion in CoO, the effective orbital angular momentum $L = 1$ can be used; see [1, 3]. Other multiplets have energies at least hundreds of terahertz (here and below we state energies in terahertz) and they do not contribute directly to the coupled spin–orbit excitations of interest. Their role (as for magnets with fully quenched orbital angular momentum) is in the generation of an effective spin anisotropy; see the discussion of the form of the effective Hamiltonian (Supplementary Equation (1)).

For a theoretical description of CoO, we introduce the $S = 3/2$ spin operator $\hat{\mathbf{S}}$ and the $L = 1$ (effective) orbital operator $\hat{\mathbf{L}}$ for any cobalt atom. The commonly used part of the Hamiltonian for CoO includes the isotropic exchange interaction for all pairs of neighbouring spins belonging to the two distinct sublattices and the exchange integral $J\hat{\mathbf{S}}_1\hat{\mathbf{S}}_2$, with $J > 0$. In addition, the effective spin–orbit interaction $\lambda\hat{\mathbf{S}}\hat{\mathbf{L}}$ with a positive constant λ and a uniaxial crystal field resulting from a tetragonal distortion of the form $-\bar{C}(\hat{L}_{[001]})^2$, $\bar{C} > 0$ are included for any Co atom; see [1, 4]. The values for these quantities are of the same order of magnitude, $\lambda = 4.53$ THz, $SZJ = 4.73$ THz with $Z = 6$ being the number of neighbours from the different sublattices and $\bar{C} = 4.26$ THz; see [4, 5].

Given the above model, the ground state is a collinear antiferromagnet, with saturated values for both orbital and spin angular momenta for any sublattice $\mathbf{S}_{1,2} = \langle\hat{\mathbf{S}}_{1,2}\rangle = \pm(3/2)\mathbf{e}_{[001]}$ and $\mathbf{L}_{1,2} = \langle\mathbf{L}_{1,2}\rangle = \mp\mathbf{e}_{[001]}$, where $\langle ... \rangle$ denotes the quantum expectation value of the operator and $e_{[001]}$ the unit vector parallel to the crystal axis [001]. For either sublattice, orbital and spin angular momenta are antiparallel.

Indeed, the CoO has lower magnetic symmetry than that appearing from the simple model described above; the sublattice magnetic moments are inclined from the symmetrical direction [001] by angles $\pm\rho$ towards the axis [110]. These directions are believed to be collinear to $[\bar{1}\bar{1}7]$; the value of $\sin\rho = \sqrt{2/51} \simeq 0.2$; see [6]. For an adequate description of this CoO structure, some extra terms should be accounted for. To describe the deviation of the magnetic moment from the [001] axis, an additional spin anisotropy with an easy axis parallel to [111]-axis was introduced [1]. Such an anisotropy appears for rhombohedral distortion of the lattice; whereas for monoclinic CoO, it is natural to consider a more general form of anisotropy. It also has been mentioned that partial quenching of the orbital angular momentum together with the low symmetry (monoclinic) distortion of the CoO lattice can result in high magnetic anisotropy of this compound with monoclinic symmetry [3]. As we shall see below, accounting for all contributions to the magnetic anisotropy, both the crystal field for the effective orbital angular momenta and magnetic anisotropy for spins, is necessary to adequately describe our experimental data.

The symmetry of CoO is low (monoclinic), and the general form of magnetic anisotropy includes many different terms, either quadratic like $\hat{L}^2_{[110]}$, $\hat{L}^2_{[1\bar{1}0]}$ or bilinear (off-diagonal) like $\hat{L}_{[110]}\hat{L}_{[100]}$; as in the case of spin anisotropy. To avoid discussing a large number of constants with unknown values, we introduce the following model, which accounts for the most important features of the system.

Let us define a new coordinate system with the unit vectors

$$\hat{\mathbf{z}} \equiv \left(-\frac{1}{\sqrt{2}}\sin\rho,\ -\frac{1}{\sqrt{2}}\sin\rho,\ \cos\rho\right),\ \hat{\mathbf{x}} \equiv \left(\frac{1}{\sqrt{2}}\cos\rho,\ \frac{1}{\sqrt{2}}\cos\rho,\ \sin\rho\right),\ \hat{\mathbf{y}} \equiv \left(-\frac{1}{\sqrt{2}},\ \frac{1}{\sqrt{2}},\ 0\right).$$

Assume that $\hat{\mathbf{z}}$ is the quantization axis for both spin and orbital angular momenta, and it is directed along the equilibrium magnetization. Then, in this coordinate system, the Hamiltonian takes the form that is characterized by rhombic symmetry; it can be written as the following

$$\mathscr{H} = \sum_{\mathbf{t}} \left[\lambda \hat{\mathbf{S}}_{\mathbf{t}} \hat{\mathbf{L}}_{\mathbf{t}} - C\hat{L}^2_{\mathbf{t},\,z} + B\hat{L}^2_{\mathbf{t},\,y} + K\hat{S}^2_{\mathbf{t},\,z} + B_1 \hat{S}^2_{\mathbf{t},x})\right] + J \sum_{\langle \mathbf{t},\ \mathbf{t}'\rangle} \hat{\mathbf{S}}_{\mathbf{t}} \hat{\mathbf{S}}_{\mathbf{t}'}, \tag{1}$$

where the first sum is taken over are translation vectors $\mathbf{t}$ for Co ions in both sublattices of the CoO lattice and $\langle \mathbf{t},\ \mathbf{t}'\rangle$ in the second sum denotes the pairs of neighbouring lattice sites corresponding to the different sublattices, where any pair of spins is counted only once. Here, in addition to uniaxial crystal field, the term with rhombic anisotropy (crystal field) for orbital angular momenta with the constant B and the biaxial magnetic anisotropy with the constants K and B_1 for spins are introduced. As we shall see below, all the anisotropy constants in Supplementary Equation (1) are positive, and the spin and orbital anisotropies are competing, e.g., the easy axis for spins and orbital angular momenta in the plain (001) are orthogonal. This feature is necessary for description of the observed values of frequencies for magnon modes of given symmetry; see the concrete estimates below. The values of $\bar{C}$ and C are expected to be different, because they are taken for different axis, z-axis and [001]-axis. The z-axis is assumed to be the equilibrium direction of the angular momenta and all off-diagonal terms like $\hat{L}_x\hat{L}_z$ or $\hat{S}_x\hat{S}_z$ should be absent here. The analysis of a more general model with full accounting of the monoclinic symmetry shows that the corresponding angles for spin and the orbital angular momenta, ρ_S and ρ_L, could be different, but information about these concrete values is absent in the literature. Accounting for the difference in the inclination angles does not produce any qualitative effects but complicates the calculations significantly. In particular, analytical results cannot be obtained in this case. In fact, the Hamiltonian (Supplementary Equation (1)) has rhombic symmetry, but the effects of monoclinic symmetry (which are significant for the explanation of our experimental results; see below) are accounted for by the inclination of the easy axis z from the crystalline axis [001]. We believe that the usage of such a “quasi-collinear” model gives a good description of dynamic properties of this compound.

The contribution of spin–orbit interactions to the spin anisotropy of magnets is well known. The orbital angular momentum of ions is quenched by the crystal field; that is, with the singlet (non-degenerate) lowest state of the ions. Spin–orbit interactions cause limited mixing of the lower singlet and higher multiplets. This mixing, being treated within perturbation theory, produces spin anisotropy. Kanamori [7, 8] extended this concept to magnets with ions like Co^{2+} or Fe^{2+} with a non-degenerate (triplet) lowest state. He found that the full orbital angular momentum of these ions ($L_{\text{free}} = 3$ for a free ion) being subject to the cubic-symmetry crystal field leads to two features: three-fold degeneracy of the lower state described by an additional variable with effective orbital angular momentum $L = 1$, and higher multiplets contributing to both orbital and spin anisotropies. Later on, it was noted that CoO, which has a low-symmetry crystal field, has much higher anisotropy constants than those of standard materials with full quenching of the orbital angular momentum, even though the field of CoO is small [3].

Our goal is to calculate the spectra of the collective excitations of a coupled system of spins and orbital angular momenta governed by the Hamiltonian (Supplementary Equation (1)). To describe the experimental data, we need to consider the collective oscillations (magnon modes) with wave

vectors $\mathbf{k}=0$. The standard approach to this problem is based on the introduction of the magnon creation and annihilation operators, $a^\dagger \propto S_x + iS_y$ and $a \propto S_x - iS_y$, respectively, and on reducing the Hamiltonian to a bilinear form in $a^\dagger$ and a taken on different lattice sites. The corresponding equations of motion contain the terms linear in $a^\dagger$ and a operators only. Note that this kind of theory can be classified as a SU(2) coherent-state theory; it is also known as the spin coherent-state theory; see [9, 10] for details. This theory required only quantum expectation values of the spin components, and it is equivalent to the Landau–Lifshitz equation, which is a closed equation for magnetization alone [9, 10]. Thus, these two approaches are equivalent and are well suited to treat Heisenberg magnets in which the single-ion anisotropy is weak and exchange interactions dominate.

In contrast, for magnets with single-ion anisotropy (for example, as with crystal-field energy for orbital angular momentum) of comparable size to the exchange interaction or spin–orbit interaction, the aforementioned approaches fail. The anisotropy term with L_x^2 is a combination of the terms $(L_x \pm iL_y)^2$. These terms lead to transitions between the single-ion states $L_z = 1$ and $L_z = -1$; i.e., a change of the z-component of the angular momentum by two. Thus, the full description of the system requires that not only dipolar variables like $\mathbf{L} = \langle \hat{\mathbf{L}} \rangle$, like for the Landau–Lifshitz equation, but also the quadrupolar variables, $L_{lk} = \frac{1}{2}\langle \hat{L}_l \hat{L}_k + \hat{L}_k \hat{L}_l \rangle$, which represent the expectation values of the operators and are bilinear in the components of $\hat{\mathbf{L}}$ on the same site, are taken into account. This specific feature was mentioned in the first work on CoO, where the full set of operators $a^\dagger$ and a for all transitions between the single-ion states was used; e.g., see Refs. [1, 11]. Of course, this analysis is quite complicated and the calculations were performed numerically.

Indeed, the same problem appears for spin-one magnets with a high non-Heisenberg interaction, single-ion anisotropy, and/or biquadratic exchange; e.g., see [12, 13, 14] and references therein. To solve this problem, we used the so-called SU(3) coherent states theory, which accounts for all possible transitions for the spin-one state and works with both dipolar and quadrupolar spin variables. The adoption of this approach used for spin-one magnets to the $L = 1$ orbital angular momentum is straightforward [15]. This approach allows the description of the full dynamics of the angular momentum-one system used in our theory to represent the effective orbital angular momentum $L = 1$.

Within this approach, the evolution of the system is described by a full set of quantum state vectors for any orbital angular momentum-one $L = 1$ operator for different sites of CoO lattice. For the given site, the state vector $|\Psi\rangle$ takes the form $|\Psi\rangle = Z_{-1}|-1\rangle + Z_0|0\rangle + Z_{+1}|+1\rangle$, where Z_{-1}, Z_0, Z_{+1} belong to the complex projective space CP(2) and $|-1\rangle$, $|0\rangle$ and $|+1\rangle$ represent the standard complete set of quantum states with given projections, -1, 0, and $+1$, respectively, along the quantization axis $\hat{\mathbf{e}}_3$ having arbitrary direction with $\hat{\mathbf{e}}_3^2 = 1$. The quantum state on one site can be parameterized by four angular variables, two for the direction of $\hat{\mathbf{e}}_3$ and two others, μ and γ, for the quadrupolar variables. The nontrivial quadrupolar variables in the moving (arbitrary-oriented) frame $\mathbf{e}_L \equiv \mathbf{e}_3$ and the unit vectors $\mathbf{e}_1, \mathbf{e}_2$, perpendicular to $\mathbf{e}_3$, are $\langle (\hat{L}_1 + i\hat{L}_2)^2 \rangle = \cos 2\mu \exp(2i\gamma)$, $\langle (\hat{L}_3 + i\hat{L}_1)^2 \rangle = \langle (\hat{L}_3 + i\hat{L}_2)^2 \rangle = 0$, and the modulus of the vector $\mathbf{L}$, $|\mathbf{L}| = \sin 2\mu < 1$. Thus, within this approach, an additional quenching of the effective orbital angular momentum in the ground state, determined by $|\langle \hat{\mathbf{L}} \rangle| = \sin 2\mu < 1$, appears naturally.

Higher momenta such as spin $S = 3/2$ also can be considered within the common approach, specifically, using the SU(4) coherent-state approach; see [16], but they are much less sensitive to these quantum effects [13]. For the description of spin degrees of freedom in our model, the phenomenological approach, where spin states are fully determined by the vector $\langle \hat{\mathbf{S}} \rangle = \mathbf{S} = \frac{3}{2}\mathbf{e}_S$, with $\mathbf{e}_S^2 = 1$, is used here. Finally, at zero temperature and within the mean-field approximation the en-

ergy of the system equals the quantum expectation value of the Hamiltonian, $\langle\hat{H}\rangle = W(\mathbf{e}_S, \mathbf{e}_L, \mu, \gamma)$. Minimizing this energy for the specified Hamiltonian (Supplementary Equation (1)) results in the value $\gamma = 0$ and the value of the orbital angular momentum $\langle\hat{\mathbf{L}}\rangle = \bar{L}\mathbf{e}_L$, which is reduced in the ground state by factor $\sin 2\mu$,

$$\bar{L} \equiv \sin 2\mu = \frac{2\lambda S}{\sqrt{4\lambda^2 S^2 + B^2}}. \tag{2}$$

Hence the "rhombic" constant B is responsible for the quantum reduction (quenching) of the orbital angular momentum. It also plays an essential role in determining the frequencies of the "longitudinal" oscillations (oscillation of the modulus of the orbital angular momentum); see Supplementary Note 2.

Supplementary Note 2: Magnon frequencies

Within the aforementioned approach, either the quenching of the orbital angular momentum in the ground state, $|\langle\hat{\mathbf{L}}\rangle| = \sin 2\mu < 1$ or the additional magnetic modes, corresponding to the longitudinal oscillations of $\langle\hat{\mathbf{L}}\rangle$, which are described by conjugated Hamilton variables $\sin 2\mu$ and γ, appear naturally. For details, see the calculations for a magnet with ferromagnetic interaction [14, 15]. In contrast, the dynamics of the spin degrees of freedom is transverse and is fully determined by the vector $\langle\hat{\mathbf{S}}\rangle = \mathbf{S} = \frac{3}{2}\mathbf{e}_S$ within the standard Landau–Lifshitz framework with the classical vector $\mathbf{S}$.

It is straightforward to show that for the approximation used (quasi-collinear model), the Hamiltonian describing the small oscillations splits into a sum of longitudinal and transverse parts and the longitudinal and transverse dynamics may be studied separately. The frequency of the longitudinal mode $\Omega_{||}$ is determined by

$$\hbar\Omega_{||} = \sqrt{4\lambda^2 S^2 + B^2} \tag{3}$$

If the orbital angular momentum is partially unquenched ($|\langle\hat{\mathbf{L}}\rangle| < 1$), this frequency exceeds the effective value of the spin–orbit interaction $2\lambda S$.

Transverse magnon modes are in fact the coupled oscillations of spin and orbital degrees of freedom. Transverse dynamics of both angular momenta are described by the classical vectors of constant length $\mathbf{S}_1$, $\mathbf{S}_2$, $\mathbf{L}_1$, $\mathbf{L}_2$ with a fixed value for parameter μ, which determines the reduction of the modulus of the orbital angular momentum in the ground state. It is more convenient to use the irreducible combinations $\mathbf{S}_{1,2}$ and $\mathbf{L}_{1,2}$, corresponding to the different sublattices,

$$\mathbf{M}_S = \mathbf{S}_1 + \mathbf{S}_2,\ \mathbf{N}_S = \mathbf{S}_1 - \mathbf{S}_2,\ \mathbf{M}_L = \mathbf{L}_1 + \mathbf{L}_2,\ \mathbf{N}_L = \mathbf{L}_1 - \mathbf{L}_2, \tag{4}$$

which are subject to constraints

$$\mathbf{M}_S^2 + \mathbf{N}_S^2 = 4S^2 = 9,\ \mathbf{M}_L^2 + \mathbf{N}_L^2 = 4\langle\hat{\mathbf{L}}\rangle^2 = 4\sin^2 2\mu,\ (\mathbf{M}_S \cdot \mathbf{N}_S) = 0,\ (\mathbf{M}_L \cdot \mathbf{N}_L) = 0. \tag{5}$$

Here the pairs $\mathbf{M}_S$, $\mathbf{M}_L$ and $\mathbf{N}_S$, $\mathbf{N}_L$ correspond to the total spin and orbital angular momenta and spin and orbital antiferromagnetic vectors, respectively. To describe small oscillations of the angular momenta, we introduce the vectors $\mathbf{N}_{S,L}$, $\mathbf{M}_{S,L}$ through their values in the ground state and the small deviations from the ground state as follow

$$\mathbf{N}_S = \mathbf{N}_S^0 + \mathbf{n}_S,\ \mathbf{N}_L = \mathbf{N}_L^0 + \mathbf{n}_L,\ \mathbf{N}_S^0 = 2S\hat{\mathbf{z}},\ \mathbf{N}_L^0 = -2\sin 2\mu\hat{\mathbf{z}},\ \mathbf{M}_{S,L} = \mathbf{m}_{S,L},$$

where we have used $\mathbf{M}=0$ in the ground state. Then the full set of equations of motion for the spin and orbital angular momenta within the linear approximation over $\mathbf{m}_{S,L}$ and $\mathbf{n}_{S,L}$ can be written

$$\hbar\frac{d\mathbf{m}_\nu}{dt}=[\mathbf{N}_\nu^0\times\mathbf{h}_\nu^{\text{eff}}],\ \ \hbar\frac{d\mathbf{n}_\nu}{dt}=[\mathbf{N}_\nu^0\times\mathbf{H}_\nu^{\text{eff}}], \tag{6}$$

where the indexes $\nu=S$ or $\nu=L$ are used for spin or orbital vectors, respectively. Here the effective fields are introduced in the standard way,

$$\mathbf{H}_\nu^{\text{eff}}=-\frac{\partial W}{\partial\mathbf{m}_\nu},\quad \mathbf{h}_\nu^{\text{eff}}=-\frac{\partial W}{\partial\mathbf{n}_\nu}, \tag{7}$$

where $W=\langle\Psi|\mathscr{H}|\Psi\rangle$ is the energy of the system, which equals the quantum expectation value of the Hamiltonian over the general SU(3) coherent states with the equilibrium values of the parameters μ and γ. The energy W includes contributions from both the magnetic energy W^{magn} and the (time-varying) interaction of the magnetic system with light W^{MO}. The latter is linear over the components of $\mathbf{m}_\nu$, $\mathbf{n}_\nu$, an aspect to be discussed in Supplementary Note 3. With the use of constraint (Supplementary Equation (5)), the magnetic energy can be written as quadratic in the components of the small vectors $\mathbf{m}_\nu$ and $\mathbf{n}_\nu$,

$$W^{\text{magn}}=\frac{A_1}{4\bar{L}}(m_{L,y}^2+n_{L,y}^2)+\frac{A_2}{4\bar{L}}(m_{L,x}^2+n_{L,x}^2)+\frac{A_3}{4S}n_{S,x}^2+\frac{A_4}{4S}m_{S,y}^2 \\ +\frac{A_5}{4S}m_{S,x}^2+\frac{A_6}{4S}n_{S,y}^2+\frac{1}{2}\lambda(\mathbf{m}_L\mathbf{m}_S+\mathbf{n}_L\mathbf{n}_S), \tag{8}$$

where to simplify the equations the notation

$$A_1=(B+C)\cot\mu+\lambda S,\ A_2=C\tan\mu+\lambda S,\ A_3=2S(B_1+K)+\lambda\bar{L}, \\ A_4=2S(K+6J)+\lambda\bar{L},\ A_5=2S(B_1+K+6J)+\lambda\bar{L},\ A_6=2KS+\lambda\bar{L}, \tag{9}$$

are used. Thus the effective fields are linear over the components of $\mathbf{m}_\nu$, $\mathbf{n}_\nu$ and they equal zero in the ground state.

Note that Supplementary Equations (6)-(7) have the same form as the classical Landau–Lifshitz equations written for $\mathbf{m}_\nu$, $\mathbf{n}_\nu$; see, e.g., Eqs. (2,3) in Ref. [17] with zero values for the effective fields in the ground state. The quantum effects typical of general SU(3) models manifests themselves only through the renormalization of the parameters determining energy W^{magn}, which differs following a naive substitution of classical vectors $\mathbf{L}_{1,2}$ into the Hamiltonian (Supplementary Equation (1)). The restoration of the Landau–Lifshitz equations, as well as the simple uncoupling of the longitudinal and transverse degrees of freedom, is valid for linear oscillations only, otherwise the equations are much more complicated. Indeed, all our experimental data show that the linear regime is realized, and the above equations are valid in our case.

The vectorial Supplementary Equation (6) can be rewritten as the set of eight equations for the x- and y-components of $\mathbf{m}_S$, $\mathbf{m}_L$, $\mathbf{n}_S$ and $\mathbf{m}_L$. Normal modes of the system are described by the free solutions of this set, valid outside the time interval for the action of the pulse. This set of equations is split as two uncoupled systems of equations. One describes the oscillations with symmetry Γ_1,

$$\hbar\frac{dm_{L,y}}{dt}=A_2n_{L,x}+\lambda\bar{L}n_{S,x},\ \hbar\frac{dn_{L,x}}{dt}=-A_1m_{L,y}-\lambda\bar{L}m_{S,y}, \\ \hbar\frac{dn_{S,x}}{dt}=A_4m_{S,y}+\lambda Sm_{L,y},\ \hbar\frac{dm_{S,y}}{dt}=-A_3n_{S,x}-\lambda Sn_{L,x}, \tag{10}$$

and the other corresponds to the symmetry Γ_2,

$$\hbar\frac{dn_{L,y}}{dt} = A_2 m_{L,x} + \lambda\bar{L} m_{S,x},\ \hbar\frac{dm_{L,x}}{dt} = -A_1 n_{L,y} - \lambda\bar{L} n_{S,y},$$
$$\hbar\frac{dm_{S,x}}{dt} = A_6 n_{S,y} + \lambda S n_{L,y},\ \hbar\frac{dn_{S,y}}{dt} = -A_5 m_{S,x} - \lambda S m_{L,x}. \quad (11)$$

As expected, the orbital and spin components like the pairs $m_{S,x}$ and $m_{L,x}$, $n_{S,x}$ and $n_{L,x}$, $m_{S,y}$ and $m_{L,y}$, and $n_{S,y}$ and $n_{L,y}$ have the same symmetry and correspond to the same mode. The solution can be found by the standard exponential ansatz, e.g., $m_{S,x} = \mathrm{Re}\{\mathscr{A}_{m,S,x}\exp(i\Omega t)\}$, where all $\mathscr{A}$'s are complex amplitudes and Ω's are the frequencies for the different modes of oscillation.

We begin with the Γ_1 oscillations. There are two frequencies for this symmetry $\Omega_{1,\pm}$, which are determined by the equation

$$\hbar^2\Omega^2_{\Gamma_1,\pm} = \frac{1}{2}[A_1A_2 + A_3A_4 - 2\lambda^2 S\bar{L} \pm \sqrt{(A_1A_2 - A_3A_4)^2 - 4\lambda^2 S\bar{L}(A_1 - A_3)(A_2 - A_4)}] \quad (12)$$

All other characteristics of the given mode can be found as well, for example, the ratio of the amplitudes of the oscillation of spin and orbital angular momenta, $\mathscr{A}_{n,L}/\mathscr{A}_{n,S}$, is found to be

$$\left(\frac{\mathscr{A}_{n,L}}{\mathscr{A}_{n,S}}\right)_{\Gamma_1,\pm} = \frac{\lambda\bar{L}(A_1 - A_3)}{\hbar^2\Omega^2_{\Gamma_1,\pm} + \lambda^2 S\bar{L} - A_1A_2} \quad (13)$$

Analogously, for the Γ_2 modes, the frequencies are

$$\hbar^2\Omega^2_{\Gamma_2,\pm} = \frac{1}{2}[A_1A_2 + A_5A_6 - 2\lambda^2 S\bar{L} \pm \sqrt{(A_1A_2 - A_5A_6)^2 - 4\lambda^2 S\bar{L}(A_1 - A_5)(A_2 - A_6)}] \quad (14)$$

and

$$\left(\frac{\mathscr{A}_{n,L}}{\mathscr{A}_{n,S}}\right)_{\Gamma_2,\pm} = \frac{\lambda\bar{L}(A_2 - A_6)}{\hbar^2\Omega^2_{\Gamma_2,\pm} + \lambda^2 S\bar{L} - A_1A_2} \quad (15)$$

Hence there are two sets of modes of a given pair of symmetries, Γ_1 and Γ_2, and within either set there are two modes that differ by their frequencies and the ratios of the amplitudes of the oscillations of spin and orbital angular momenta. The analysis shows that for both symmetries, Γ_1 and Γ_2, the modes with the lower frequencies have smaller relative amplitudes of oscillation of the orbital angular momentum than for the spin angular momentum. Hence it is natural to refer to the low-frequency modes $\Omega_{\Gamma_{1,2},-}$ as spin-dominated and to refer to the high-frequency modes $\Omega_{\Gamma_{1,2},+}$ as orbital-dominated. Below we use the following notation:

$$\Omega_{\Gamma_1,-} \equiv \Omega_{1,S},\ \Omega_{\Gamma_1,+} \equiv \Omega_{1,L},\ \Omega_{\Gamma_2,-} \equiv \Omega_{2,S},\ \Omega_{\Gamma_2,+} \equiv \Omega_{2,L}.$$

According to the experimental data, the lower modes with frequencies 4.4 THz and 6.6 THz were attributed to symmetries Γ_2 and Γ_1, respectively. The high-frequency modes for both symmetries were considered to be degenerated with both frequencies equal to 8.9 THz. This data can be used to find the parameters entering the Hamiltonian (Supplementary Equation (1)). The formulae for the frequencies contain six constants, whereas only four frequencies are known. The analysis shows that for the parameter values determined in the previous article [4, 5], $\lambda = 4.5$ THz, $C = 4.26$ and $J = 0.53$ THz, the frequencies are noticeably higher than those observed in our experiment (the lower frequency calculated with $C = 4.2$ was 5.4 THz). We found that our data cannot

be fitted for $\lambda > \lambda_C = 4.5$ THz or $J > J_C = 0.48$ THz. Therefore we need to use lower values for the spin–orbit constant λ and the exchange integral J than has been used before [4, 5]. To describe the experimental data, we choose different values for λ and J; the constant C and the additional parameters in Supplementary Equation (1), B, B_1 and K were found to fit the observed values of the frequencies. For values $\lambda = 4.2$ THz and $J = 0.45$ THz, the constants yielded: $C = 2.811$, $B = 1.013$ and $B_1 = 0.996$, with a very small $K = 0.096$ (all values given in THz).

The classification of the modes as spin-dominated and orbital-dominated is valid for all modes, however the ratios of the amplitudes of spin and orbital oscillations are not too large. For example, for the above set of parameters, the value of $(\mathscr{A}_{n,L}/\mathscr{A}_{n,S})$ equals 0.443 and 0.649 for Γ_2 (4.4 THz) and Γ_1 (6.6 THz) low-frequency modes, respectively. For the high-frequency Γ_2 and Γ_1 modes (degenerate with frequency 8.9 THz), the value for the ratio $(\mathscr{A}_{n,L}/\mathscr{A}_{n,S})$ equals 1.498 and 1.175 and the value for the ratio $(\mathscr{A}_{m,L}/\mathscr{A}_{m,S})$ equals 1.03 and 1.5, respectively.

It turns out that the results of the above analysis appear to be quite stable against variations of the initial values of λ and J within reasonably wide intervals far from the aforementioned critical values. For all pairs of parameters in the intervals (4.3 - 4.0) THz for λ and (0.45 - 0.4) THz for J the values of parameters B and B_1 remain of the order of 1 THz and K does not exceed 0.1 THz.

Note the competing character of the spin and orbital anisotropy. The positive values of the constant B_1 and B, describing the orthogonal easy axis in the basal plane for spin and orbital angular momenta, appear naturally to explain the experimental observation, specifically, that the mode with lowest frequency (4.4 THz) has the lower symmetry Γ_2.

The important feature of the estimates is that the splitting of lower modes is quite large, whereas the higher modes are, within the experimental accuracy, degenerate. This is understood based on the polarization analysis of the modes. First note that for antiferromagnets with weak anisotropy, the amplitude of the oscillations of total magnetic moment **m** is much smaller than for antiferromagnetic vector **n**. In contrast, for CoO, our calculation shows that the ratio of the amplitude of the oscillations of the components of **m** and **n** is small (equal to 0.23 and 0.25 for spin and orbital vectors) only for the 4.4-THz mode. For the 6.6-THz mode these values are 0.42 and 0.37, respectively, whereas for higher frequencies the amplitudes of **n** are even smaller than the amplitudes of **m** by a factor of 0.7–0.8. For a given mode, the vectors **m** and **n** are oscillating in perpendicular directions. Therefore, the high-frequency orbital-dominated modes are less sensitive to the rhombic anisotropy. Note as well that the low-frequency spin-dominated modes are more strongly sensitive to the spin anisotropy (determined by the constant B_1) than to orbital anisotropy (determined by the constant B). All these factors give a qualitative explanation for the general features found by the analysis of the equations for frequencies.

Supplementary Note 3: Interaction between light and magnons

The standard explanation of light-induced magnon excitation and their observation is based on the phenomenological treatment of the magneto-optical Faraday effect (FE) and the Cotton–Mouton effect (CME) and their corresponding inverse effects (IFE and ICME). The interaction of light with a transparent magnetic medium is described by the Hamiltonian $\mathscr{H}^{\mathrm{MO}}$ [18],

$$\mathscr{H}^{\mathrm{MO}}(t) = -\frac{\delta\varepsilon_{ij}}{16\pi}\mathscr{E}_i(t)\mathscr{E}_j^*(t), \tag{16}$$

where $\mathscr{E}_i(t)$ is the time-dependent complex amplitude of the optical electric field $E_i(t) = \mathrm{Re}[\mathscr{E}_i(t)e^{i\omega t}]$ and $\delta\varepsilon_{ij}$ is the part of dielectric permittivity tensor, dependent on the magnetic state of the medium.

The phenomenological form of the tensor $\delta\varepsilon_{ij}$ can be constructed with the expansion over components of the angular momentum, accounting for the magnetic symmetry and Onsager relation; see, e.g., [18]. For antiferromagnets, this expansion includes the components of the total ferromagnetic vector $\mathbf{M}$ and antiferromagnetic vector $\mathbf{N}$. For the all-optical pump-probe method, magnon excitation and the signal created by magnons can be described solely in terms linear over small oscillations, i.e., linear on the components of $\mathbf{m}$ and $\mathbf{n}$. For magnets like CoO, where the Dzyaloshinskii–Moriya interaction is forbidden by symmetry, this expansion takes the form

$$\delta\varepsilon_{ij} = ik_{ijk}m_k + 2g_{ijkl}N_k n_l, \tag{17}$$

where k_{ijk} and g_{ijkl} determine the FE and the CME (both direct and inverse), respectively. Here $k_{ijk} = -k_{jik}$, $g_{ijkl} = g_{jikl} = g_{ijlk}$, the structures of these tensors are determined by the magnetic symmetry group of the crystal [19, 20]. Note that the subscript "l" is not related to the orbital angular momentum, but a lower index for a tensor "$ijkl$".

As a rule, the microscopic origin of the interaction for visible and near-infrared light is the electric dipole transitions, associated with the orbital angular momentum. However, for many compounds with iron-group transition elements, such as orthoferrites and iron borate, the orbital angular momentum of the atom is considered to be quenched by the crystal field. To describe magneto-optical effects, it is necessary to take into account the orbital degrees of freedom (indeed, weakly-unquenched by spin–orbit interaction), which play the role much like slave variables in following the dynamics of spins; see a concrete analysis in the recent article [21] and references therein. In this approximation, the magnets and their interaction with light can be described by an effective spin Hamiltonian, and $\mathbf{m}$ and $\mathbf{n}$ represent spin ferromagnetic and antiferromagnetic vectors, respectively.

For materials like CoO with partially unquenched orbital angular momentum, the independent dynamics of the spin and orbital angular momenta should be considered. Formally, the independent contributions of these angular momenta, $\mathbf{m}_S$, $\mathbf{n}_S$ or $\mathbf{m}_L$, $\mathbf{n}_L$ can be written down in the form given in Supplementary Equation (17). As was shown above, the symmetric properties of the oscillations of the corresponding spin and orbital vectors are the same, as well as the symmetric properties of the tensors k_{ijk} and g_{ijkl}. Although both the orbital and spin angular momenta contribute to the magnetism in CoO, the magneto-optical effect is dominated by the orbital angular momentum because the optical selection rule for the electric-dipole interaction only allows changes in orbital angular momentum. A change in spin can be only allowed via spin–orbit interactions. In many other systems, however, the orbital angular momentum is quenched and cannot contribute to the magnetic properties of media, and thus magneto-optical effect is governed by the indirect coupling between spin and light. To simplify the expressions in this section further, we omit the indexes "L" from vectors $\mathbf{m}_L$, $\mathbf{n}_L$.

Now consider the torque produced by light pulses in the magnetic system. The effective energy of the magneto-optical interaction $W^{\text{MO}} = \langle\Psi|\mathscr{H}^{\text{MO}}|\Psi\rangle$, with the use of Supplementary Equation (17) for both TG and LG, can be written as:

$$W^{\text{MO}}_{\text{TG}}(t) = -\frac{I(t)}{16\pi}[(G_1+G_2+G_3)n_x + (G_1-G_2-G_3)n_x\cos 2\theta - G_4 n_y(1-\cos 2\theta) \\ + (G_5 n_x + G_6 n_y)\sin 2\theta\cos\psi + (K_1 m_y - K_2 m_x)\sin 2\theta \sin\psi], \tag{18}$$

$$W^{\mathrm{MO}}_{\mathrm{LG}}(t) = -\frac{I(t)}{16\pi}[(G_2+G_3)n_x + 2G_4 n_y \cos 2\theta + (G_2-G_3)n_x \sin 2\theta \cos\psi + K_3 m_x \sin 2\theta \sin\psi], \quad (19)$$

where $I(t) = \mathscr{E}(t)\mathscr{E}^*(t)$ for a given polarization of light. Here, the significant combinations of the tensor components g_{ijkl} and k_{ijk}, G and K, are introduced:

$$G_1 = 2N(g_{xxzx}\sin^2\rho + g_{zzzx}\cos^2\rho + g_{zxzx}\sin 2\rho), \quad (20)$$

$$G_2 = N(g_{xxzx}\cos^2\rho + g_{zzzx}\sin^2\rho - g_{zxzx}\sin 2\rho), \quad (21)$$

$$G_3 = g_{yyzx}N, \quad (22)$$

$$G_4 = N(g_{yzyz}\sin\rho - g_{xyyz}\cos\rho), \quad (23)$$

$$G_5 = \sqrt{2}N[(g_{xxzx} - g_{zzzx})\sin 2\rho + g_{zxzx}\cos 2\rho], \quad (24)$$

$$G_6 = \sqrt{2}N(g_{yzyz}\cos\rho + g_{xyyz}\sin\rho), \quad (25)$$

$$K_1 = \frac{k_{zxy}}{\sqrt{2}}, \quad (26)$$

$$K_2 = \frac{(k_{yzx}\cos\rho - k_{xyx}\sin\rho)}{\sqrt{2}}, \quad (27)$$

$$K_3 = k_{yzx}\sin\rho + k_{xyx}\cos\rho. \quad (28)$$

Note that the only components g_{zxzx}, g_{yzyz} and k_{yzx} or k_{zxy} are non-zero for the cubic symmetry of the tensors g_{ijkl} and k_{ijk}. All other components, namely, g_{xxzx}, g_{zzzx}, g_{yyzx}, g_{xyyz} for the CME and k_{xyx} for the FE, are caused exclusively by low (monoclinic) symmetry of these tensors.

Recall that the angular variables θ and ψ here determine the polarization of the pump light. The values $\psi = \mp 90^\circ$ with $\theta = 45^\circ$ correspond to two alternative circular polarizations, $\sigma_\pm$, whereas $\psi = 0^\circ$ describes the linear polarization of light with the polarization plane inclined at an angle θ to some crystalline axis ([001] for the TG and [100] for the LG).

From Supplementary Equations (18) and (19), the effective magnetic field pulses can be defined as $H_{x,y} = -\partial W^{\mathrm{MO}}/\partial m_{x,y}$ and $h_{x,y} = -\partial W^{\mathrm{MO}}/\partial n_{x,y}$ [22]. These effective magnetic fields are acting as the driving forces for the excitation of magnons.

Supplementary Equations (10) and (11) for either geometry, TG or LG, can be rewritten as linear equations with right-hand side parts describing the driving force exciting the magnons, $\frac{dm_{x,y}}{dt}|_{RHS}$ and $\frac{dn_{x,y}}{dt}|_{\mathrm{RHS}}$. In the TG, magnons with Γ_1 are triggered as

$$\left.\frac{dm_y^{\mathrm{TG}}}{dt}\right|_{\mathrm{RHS}} = -\frac{\gamma N I(t)}{16\pi}[(G_1+G_2+G_3) + (G_1-G_2-G_3)\cos 2\theta + G_5 \sin 2\theta \cos\psi] \quad (29)$$

$$\left.\frac{dn_x^{\mathrm{TG}}}{dt}\right|_{\mathrm{RHS}} = -\frac{\gamma N I(t)}{16\pi} K_1 \sin 2\theta \sin\psi, \quad (30)$$

whereas the excitation of the magnons with Γ_2 is described by the equations

$$\left.\frac{dm_x^{\mathrm{TG}}}{dt}\right|_{\mathrm{RHS}} = -\frac{\gamma N I(t)}{16\pi}[G_4(1-\cos 2\theta) + G_6 \sin 2\theta \cos\psi] \quad (31)$$

$$\left.\frac{dn_y^{\rm TG}}{dt}\right|_{\rm RHS} = \frac{\gamma NI(t)}{16\pi} K_2 \sin 2\theta \sin \psi. \tag{32}$$

Analogously, for the LG, the excitation of magnons with Γ_1 is described by the equations

$$\left.\frac{dm_y^{\rm LG}}{dt}\right|_{\rm RHS} = -\frac{\gamma NI(t)}{16\pi} [(G_2 + G_3) + (G_2 - G_3) \sin 2\theta \cos \psi] \tag{33}$$

$$\left.\frac{dn_x^{\rm LG}}{dt}\right|_{\rm RHS} = 0, \tag{34}$$

whereas the excitation of magnons with Γ_2 is governed by

$$\left.\frac{dm_x^{\rm LG}}{dt}\right|_{\rm RHS} = \frac{\gamma NI(t)}{8\pi} G_4 \cos 2\theta \tag{35}$$

$$\left.\frac{dn_y^{\rm LG}}{dt}\right|_{\rm RHS} = -\frac{\gamma NI(t)}{16\pi} K_3 \sin 2\theta \sin \psi. \tag{36}$$

Supplementary Note 4: Efficiency of magnon excitation

We compared the efficiencies of the magnon excitations in orbital-unquenched CoO and orbital quenched NiO [23]. The magnon oscillation amplitudes were normalized using the pump fluence.

The magnon frequencies up to 4 THz are not negligible compared with the inverse of the pump and probe pulse durations. Therefore, these pulses are not regarded as ideal delta functions, but as Gaussian functions with finite duration. This effect leads to a decrease of the excitation efficiency and can be incorporated with the spectral weight of the Gaussian pulse;

$$W(\omega) = \exp\left[-4\ln 2 \frac{\omega^2}{\Delta\omega^2}\right]. \tag{37}$$

Here, $\Delta\omega = 4\ln 2/\Delta t$, where Δt is the pulse duration (FWHM) of the pump and probe pulses, and ω is the angular frequency of the magnon.

For NiO: $\omega/2\pi = 1.07$ THz, $\Delta t_{\rm probe} = 90$ fs, $\Delta\omega_{\rm probe}/2\pi = 4.9$ THz, $W(\omega)_{\rm probe} = 0.9$, $\Delta t_{\rm pump} = 50$ fs, $\Delta\omega_{\rm pump}/2\pi = 8.8$ THz, $W(\omega)_{\rm pump} = 1.0$, peak-to-peak amplitude of the oscillation $A = 0.5$ mrad, pump fluence $I_{\rm pump} = 80$ mJ cm^{-2}.
For CoO: $\omega/2\pi = 4.4$ THz, $\Delta t_{\rm probe} = \Delta t_{\rm pump} = 50$ fs, $\Delta\omega/2\pi = 8.8$ THz, $W(\omega)_{\rm probe} = W(\omega)_{\rm pump} = 0.5$, $A = 50$ mrad, $I_{\rm pump} = 130$ mJ cm^{-2}.

It is reasonable to define the efficiency of the magnon excitation η as;

$$\eta = \frac{A}{I_{\rm pump} \times W(\omega)_{\rm pump} \times W(\omega)_{\rm probe}} \tag{38}$$

Therefore, the ratio of the efficiency of η between CoO and NiO is

$$\frac{\eta(\mathrm{CoO})}{\eta(\mathrm{NiO})} = \frac{\dfrac{50}{0.5 \times 0.5 \times 130}}{\dfrac{0.5}{0.9 \times 1.0 \times 80}} \simeq 200 \tag{39}$$

In the two-color pump-probe experiment, the birefringence suppresses the output-to-input ratio depending on the pump and probe wavelengths and sample thickness [17, 24, 25]. The CoO crystal in the LG has a static birefringence of $\Delta n \simeq 0.001$ (see Methods), which leads to a phase shift of $\simeq \pi/10$ ($\ll 2\pi$) between the ordinary and extraordinary components of the pump/probe beams. Thus, one can assume that the pump beam maintains its polarization through the sample. Note that in the experiment with a NiO crystal [23], the authors chose a magnetic domain that was free from birefringence. Therefore, the presence of slight static birefringence in CoO does not influence the conclusion of the orders-of-magnitude comparison of the output-to-input ratios between the CoO (in the LG) and NiO experiments.

Supplementary References

1. Chou, H.-h. & Fan, H. Y. Light scattering by magnons in CoO, MnO, and α-MnS. *Phys. Rev. B* **13,** 3924–3938 (1976).

2. Fujii, Y., Katayama, D., & Koreeda, A. Broadband light scattering spectroscopy utilizing an ultra-narrowband holographic notch filter. *Jpn. J. Appl. Phys.* **55,** 10TC03 (2016).

3. Daniel, M. R. & Cracknell, A. P. Magnetic symmetry and antiferromagnetic resonance in CoO. *Phys. Rev.* **177,** 932–941 (1969).

4. Kant, Ch. *et al.* Optical spectroscopy in CoO: Phononic, electric, and magnetic excitation spectrum within the charge-transfer gap. *Phys. Rev. B* **78,** 245103 (2008).

5. Yamani, Z., Buyers, W. J. L., Cowley, R. A. & Prabhakaran, D. Magnetic excitations of spin and orbital moments in cobalt oxide. *Can. J. Phys.* **88,** 729–733 (2010).

6. Roth, W. L. Magnetic structures of MnO, FeO, CoO, and NiO. *Phys. Rev.* **110,** 1333–1341 (1958).

7. Kanamori, J. Theory of the magnetic properties of ferrous and cobaltous oxides I. *Prog. Theor. Phys.* **17,** 177–196 (1957).

8. Kanamori, J. Theory of the magnetic properties of ferrous and cobaltous oxides II. *Prog. Theor. Phys.* **17,** 197–222 (1957).

9. Perelomov, A. M. Generalized coherent states and some of their applications. *Sov. Phys. Usp.* **20,** 703–720 (1977).

10. Fradkin, E. *Field Theories of Condensed Matter Systems.* 2nd ed. (Cambridge University Press, 2013).

11. Alben, R. Theory of magnetic excitations in $FeCl_2$ and CoO. *J. Appl. Phys.* **40,** 1112–1113 (1969).

12. Papanicolaou, N. Unusual phases in quantum spin-1 systems. *Nucl. Phys.* **B305**, 367–395 (1988).

13. Loktev, V. M. & Ostrovskii, V. S. The peculiarities of statics and dynamics of magnetic insulators with single-ion anisotropy. *Low Temp. Phys.* **20,** 775–800 (1994).

14. Bar'yakhtar, V. G., Butrim, V. I., Kolezhuk, A. K. & Ivanov, B. A. Dynamics and relaxation in spin nematics. *Phys. Rev. B* **87,** 224407 (2013).

15. Butrim, V. I. Magnetic resonance frequencies of ferromagnets with partially frozen orbital momentum. *Low Temp. Phys.* **40,** 508–512 (2014).

16. Fridman, Yu. A., Kosmachev, O. A., Kolezhuk, A. K. & Ivanov, B. A. Spin nematic and antinematic states in a spin-3/2 isotropic non-Heisenberg magnet. *Phys. Rev. Lett.* **106,** 097202 (2011).

17. Iida, R. *et al.* Spectral dependence of photoinduced spin precession in $DyFeO_3$. *Phys. Rev. B* **84,** 064402 (2011).

18. Landau, L. D. & Lifshitz, E. M. *Electrodynamics of Continuous Media* (Pergamon, 1984).

19. Cracknell, A. P. Scattering matrices for the Raman effect in magnetic crystals. *J. Phys. C* **2,** 500–511 (1969).

20. Smolenskiĭ, G. A., Pisarev, R. V. & Siniĭ, I. G. Birefringence of light in magnetically ordered crystals. *Sov. Phys. Usp.* **18,** 410–429 (1975).

21. Battiato, M., Barbalinardo, G. & Oppeneer, P. M. Quantum theory of the inverse Faraday effect. *Phys. Rev. B* **89,** 014413 (2014).

22. Kalashnikova, A. M. *et al.* Impulsive generation of coherent magnons by linearly polarized light in the easy-plane antiferromagnet $FeBO_3$. *Phys. Rev. Lett.* **99,** 167205 (2007).

23. Tzschaschel, C. *et al.* Ultrafast optical excitation of coherent magnons in antiferromagnetic NiO. *Phys. Rev. B* **95,** 174407 (2017).

24. Woodford, S. R., Bringer, A. & Blügel, S. Interpreting magnetization from Faraday rotation in birefringent magnetic media. *J. Appl. Phys.* **101,** 053912 (2007).

25. de Jong, J. A. *et al.* Effect of laser pulse propagation on ultrafast magnetization dynamics in a birefringent medium. *J. Phys.: Condens. Matter* **29,** 164004 (2017).
